# The 18 Household Food Security Survey items provide valid food security classifications for adults and children in the Caribbean

**DOI:** 10.1186/1471-2458-6-26

**Published:** 2006-02-08

**Authors:** Martin C Gulliford, Cheryl Nunes, Brian Rocke

**Affiliations:** 1 Division of Health and Social Care Research, King's College London, London, UK; 2 Nutrition Division, Ministry of Health, Laventille, Trinidad and Tobago, UK

## Abstract

**Background:**

We tested the properties of the 18 Household Food Security Survey (HFSS) items, and the validity of the resulting food security classifications, in an English-speaking middle-income country.

**Methods:**

Survey of primary school children in Trinidad and Tobago. Parents completed the HFSS. Responses were analysed for the 10 adult-referenced items and the eight child-referenced items. Item response theory models were fitted. Item calibrations and subject scores from a one-parameter logistic (1PL) model were compared with those from either two-parameter logistic model (2PL) or a model for differential item functioning (DIF) by ethnicity.

**Results:**

There were 5219 eligible with 3858 (74%) completing at least one food security item. Adult item calibrations (standard error) in the 1PL model ranged from -4.082 (0.019) for the 'worried food would run out' item to 3.023 (0.042) for 'adults often do not eat for a whole day'. Child item calibrations ranged from -3.715 (0.025) for 'relied on a few kinds of low cost food' to 3.088 (0.039) for 'child didn't eat for a whole day'. Fitting either a 2PL model, which allowed discrimination parameters to vary between items, or a differential item functioning model, which allowed item calibrations to vary between ethnic groups, had little influence on interpretation. The classification based on the adult-referenced items showed that there were 19% of respondents who were food insecure without hunger, 10% food insecure with moderate hunger and 6% food insecure with severe hunger. The classification based on the child-referenced items showed that there were 23% of children who were food insecure without hunger and 9% food insecure with hunger. In both children and adults food insecurity showed a strong, graded association with lower monthly household income (P < 0.001).

**Conclusion:**

These results support the use of 18 HFSS items to classify food security status of adults or children in an English-speaking country where food insecurity and hunger are more frequent overall than in the US.

## Background

Food insecurity has been defined as the 'limited or uncertain availability of nutritionally adequate and safe foods, or limited or uncertain ability to acquire acceptable foods in socially acceptable ways' [1]. Severe food insecurity and hunger can lead to food intakes that are continuously insufficient to meet dietary energy requirements [2]. Less severe food insecurity is associated with reduced quality and variety of dietary intakes [3,4], possibly associated with obesity in adults [5], and a range of adverse developmental, psychological and health outcomes in children[6]. Our previous studies in Trinidad and Tobago have shown that food insecurity without hunger is common in both adults and adolescents. It is associated with markers of poorer dietary quality [7,8] and is associated with underweight in adults [7].

The assessment of food insecurity and hunger in population surveys has been facilitated by the development in the US of a standard questionnaire measure which may be used to classify the food security status of adults and children [1,9]. The 18-item food security measure is referred to as the Household Food Security Survey (HFSS) module. The 18 items include 10 which concern the experiences of adults and eight concerning respondents' experiences of providing food to children in their households [9,10]. The food security items have been included in the US Current Population Survey (CPS) since 1995. CPS data revealed that overall some 12% of US households are food insecure, including 3.3% with moderate hunger and 0.8% with severe hunger [1]. There are considerably higher proportions of food insecure households in inner-city and ethnic minority communities [1]. Children, especially younger children, are protected from the consequences of household food insecurity until this is severe. In the US, only 0.7% of households with children were classified as having hunger among children in 1998–9 [10].

In view of the potential which this instrument provides for assessing the food security status of households in the Caribbean region, we investigated whether the 18 HFSS items could be implemented successfully in an English-speaking middle-income country, Trinidad and Tobago. We specifically aimed to test the properties of the adult- and child-referenced food security items for use in Caribbean communities by fitting item response theory models and thus evaluate the validity of the resulting food security classifications. This report extends our previous observations with the short-form, six-item food security scale [7,8].

## Methods

### Subjects

We carried out a cross-sectional survey of school children in Trinidad and Tobago. There are 468 primary schools in Trinidad and Tobago, 433 in Trinidad and 35 in Tobago. The sample of 66 schools was drawn by stratifying the nation into health administrative areas and randomly selecting schools with probability proportional to size. The sample of schools was drawn by the Central Statistical Office for earlier surveys carried out in 1989 and 1999 [11]. Fieldwork was carried out in the first six months of 2004. Within each school we measured all children in the first year classes (whose fifth birthday was generally in the school year) and in the classes for children aged 8 to 9 years (whose ninth birthday was generally in the school year). Measurements were made of height, weight and skinfold thicknesses and these data will be reported elsewhere. We did not attempt to identify children living in the same households. The study received approval from the research ethics committee at the General Hospital, Port of Spain, it was also approved by the Ministry of Education, parents gave written informed consent for completion of measurements and questionnaires.

### Questionnaires

The parents of each child were asked to complete the survey questionnaire in self-completion format. According to UNICEF data, the total adult literacy rate in Trinidad and Tobago is 98%. If it was necessary the questionnaire was interview-administered by a class teacher or a fieldworker but the proportion of subjects receiving such assistance was not recorded. The questionnaire included the 18 HFSS items with the wording described by Bickel et al [9] but with appropriate adjustment for self-administration. Respondents were required to complete every item with no items skipped or screened out. As the 'balanced meal' item is known to present difficulties of interpretation, the following explanation was placed next to the response options for both the child- and adult-referenced 'balanced meal' items: 'a balanced meal may contain starchy food, like rice, potatoes, bread, ground provisions or macaroni ; and a protein-rich food like meat, fish, milk, or peas or beans; and a fruit or a vegetable'. This wording was agreed by local nutritionists working in the government health service adapted from a suggestion of Derrickson et al[12]. A pilot study was conducted to confirm that the questionnaire items were understood and could be completed successfully.

The population in Trinidad and Tobago is approximately 40% of African descent and 40% of Indian subcontinent descent, with subjects of mixed ethnicity comprising most of the remainder. Each child's ethnicity was reported by the parents using the categories 'Afro-Caribbean', 'Indo-Caribbean', 'Mixed' and 'other and not known' for analysis as described previously [11]. The child's gender and date of birth were recorded from the school register onto measurement forms. The questionnaire included an item about monthly household income using ten categories but the highest three categories were combined for analysis. Values were converted to US dollars using an exchange rate of US$1 = TT$6.

### Analysis

Item response theory models (IRT) are a family of statistical models which may be fitted to data from multi-item tests and questionnaire measures. In contrast to classical psychometric theory which emphasises scale scores, item response theory focuses on estimating the properties of each item in a measure. In the one-parameter logistic model, the probability of a subject, *s*, giving an affirmative response to item *i *is estimated from the difference between the item calibration or relative severity of the item, *β*_*i*_, and the trait level or severity of experienced food insecurity of the subject, θ_s_. Thus if the severity of food insecurity experienced by the subject is greater than the item calibration, an affirmative response is expected. Thus the probability that subject *s *affirms item *i *(that is, X_is _= 1) is given by

P(Xis=1|θs,βi)=exp⁡(θs−βi)1+exp⁡(θs−βi)
 MathType@MTEF@5@5@+=feaafiart1ev1aaatCvAUfKttLearuWrP9MDH5MBPbIqV92AaeXatLxBI9gBaebbnrfifHhDYfgasaacH8akY=wiFfYdH8Gipec8Eeeu0xXdbba9frFj0=OqFfea0dXdd9vqai=hGuQ8kuc9pgc9s8qqaq=dirpe0xb9q8qiLsFr0=vr0=vr0dc8meaabaqaciaacaGaaeqabaqabeGadaaakeaacqqGqbaucqGGOaakcqqGybawdaWgaaWcbaGaeeyAaKMaee4Camhabeaakiabg2da9iabigdaXiabcYha8HGaaiab=H7aXnaaBaaaleaacqqGZbWCaeqaaOGaeiilaWIae8NSdi2aaSbaaSqaaiabbMgaPbqabaGccqGGPaqkcqGH9aqpdaWcaaqaaiGbcwgaLjabcIha4jabcchaWjabbIcaOiab=H7aXnaaBaaaleaacqqGZbWCaeqaaOGaeyOeI0Iae8NSdi2aaSbaaSqaaiabbMgaPbqabaGccqGGPaqkaeaacqaIXaqmcqGHRaWkcyGGLbqzcqGG4baEcqGGWbaCcqqGOaakcqWF4oqCdaWgaaWcbaGaee4CamhabeaakiabgkHiTiab=j7aInaaBaaaleaacqqGPbqAaeqaaOGaeiykaKcaaaaa@5C21@

Item calibrations from the 1PL model locate an item in relation to the underlying latent construct of food security/food insecurity. Item calibrations indicate the relative severity of an item because items with lower calibrations are affirmed by subjects with lesser degrees of food insecurity than items with high calibrations [13,14]. In the differential item functioning model (DIF), the assumption that the calibration of an item is the same for all subjects is relaxed. It is then possible to estimate whether item calibrations vary systematically between different groups of subjects, for example those defined by ethnicity.

In the one parameter logistic model, all items are held to be equally discriminating and items only differ with respect to their calibrations or relative item severities. In a two parameter logistic model, an item discrimination parameter, α_i_, is introduced

P(Xis=1|θs,βi,αi)=exp⁡(αi(θs−βi))1+exp⁡(αi(θs−βi))
 MathType@MTEF@5@5@+=feaafiart1ev1aaatCvAUfKttLearuWrP9MDH5MBPbIqV92AaeXatLxBI9gBaebbnrfifHhDYfgasaacH8akY=wiFfYdH8Gipec8Eeeu0xXdbba9frFj0=OqFfea0dXdd9vqai=hGuQ8kuc9pgc9s8qqaq=dirpe0xb9q8qiLsFr0=vr0=vr0dc8meaabaqaciaacaGaaeqabaqabeGadaaakeaacqqGqbaucqGGOaakcqqGybawdaWgaaWcbaGaeeyAaKMaee4Camhabeaakiabg2da9iabigdaXiabcYha8HGaaiab=H7aXnaaBaaaleaacqqGZbWCaeqaaOGaeiilaWIae8NSdi2aaSbaaSqaaiabbMgaPbqabaGccqGGSaalcqaHXoqydaWgaaWcbaGaeeyAaKgabeaakiabcMcaPiabg2da9maalaaabaGagiyzauMaeiiEaGNaeiiCaaNaeeikaGIaeqySde2aaSbaaSqaaiabbMgaPbqabaGccqGGOaakcqWF4oqCdaWgaaWcbaGaee4CamhabeaakiabgkHiTiab=j7aInaaBaaaleaacqqGPbqAaeqaaOGaeiykaKIaeiykaKcabaGaeGymaeJaey4kaSIagiyzauMaeiiEaGNaeiiCaaNaeeikaGIaeqySde2aaSbaaSqaaiabbMgaPbqabaGccqGGOaakcqWF4oqCdaWgaaWcbaGaee4CamhabeaakiabgkHiTiab=j7aInaaBaaaleaacqqGPbqAaeqaaOGaeiykaKIaeiykaKcaaaaa@69EF@

In the two-parameter logistic model, the impact of the difference between the trait level and the item calibration on the probability of an affirmative response, is lower for less discriminating items [14].

For the present analyses, item response models were fitted without imputing missing values. Item response models were fitted using the BILOG-MG program from Scientific Software International [13] using marginal maximum likelihood (MML) estimation. Initially a one-parameter logistic model (1PL) was fitted to the data for all subjects as a single group [14]. In order to evaluate how well this model fitted the data, the constraints of the 1PL model were then relaxed in each of two ways. First, a two-parameter logistic model (2PL) was fitted in which the slope, or discrimination parameter, of the item characteristic curves was allowed to vary between items [15]. Fitting the 2PL model allowed us to evaluate whether the estimation of food security status was sensitive to varying the assumption of equal discrimination for all items [14]. In the 1PL model, subject scores are a function of the number of affirmatives or raw score and all subjects in a raw score category receive the same 1PL score. In the 2PL model subject scores depend not only on the number of affirmatives but also on which items are affirmed with a range of subject scores possible at a given raw score. Subject scores were compared for the 2PL and 1PL models by means of a box and whisker plot [16].

Secondly, a differential item functioning (DIF) model was fitted in which the item calibrations were allowed to vary between groups of subjects defined by ethnicity of the child [13]. Only the mean of the item calibrations was held constant across groups. Item calibrations were estimated after adjusting for variation in the average level of food insecurity between groups. Fitting the DIF model allowed us to evaluate whether it was reasonable to assume that calibrations of individual items were the same across groups of subjects defined by ethnicity [13]. The change in goodness of fit from DIF model as compared to the 1PL model was evaluated by means of likelihood ratio tests [13,14]. Differences in item calibrations (95% confidence intervals) were then estimated using the Afro-Caribbean group for reference.

Before estimating food security status, cases with missing values had food security status coded to missing if there were more than three adult items, or more than two child items missing. In the remaining cases, missing values were imputed following the recommendations of Bickel et al. [9]. Food security status was then coded using recommended cutpoints for children (food insecure without hunger, two to four affirmatives; food insecure with hunger, 5 or more affirmatives) and adults (food insecure without hunger, three to five affirmatives; food insecure with moderate hunger six to eight affirmatives; food insecure with severe hunger, nine or ten affirmatives). Differences between groups were evaluated in an ordinal logistic model with food security status as dependent variable, adjusting for age, sex and clustering by school.

## Results

There were 66 schools sampled with 5219 eligible students. Questionnaires were returned for 4215 (81%) subjects. After omitting cases with missing values for sex, age or all food security items there were 3,858 (74%) questionnaires remaining for analysis. These included 1850 boys and 2008 girls. There were 2087 aged 4 to <7 years (mean age 5.8 years) and 1771 aged 7 to 12 years (mean age 9.0 years). Tables 1 and 2 show data for adult- and child-referenced items respectively. Item non-response was approximately 3% for most of the items. Cronbach's alpha was 0.915 for the adult-referenced items and 0.818 for the child-referenced items respectively.

The item calibrations from the 1PL model estimate the relative location of the items in relation to the underlying latent construct of food security/food insecurity. The mean of the item calibrations was assigned a value of zero. When an item calibration has a negative sign, an affirmative response to this item denotes a relatively lower severity of food security. An affirmative response to an item with a positive sign denotes relatively higher severity of food insecurity. Item calibrations from the 1PL model ranged from -4.082 (standard error 0.019) for the low severity item concerning 'worried that food would run out' to 3.023 (0.042) for the high severity item about 'adults often did not eat for a whole day' (Table 1). Similar item calibrations were obtained from the two-parameter logistic model but the 'balanced meal' item gave a lower discrimination parameter than the other items. The difference in goodness of fit from fitting the 2PL model was χ^2 ^= 228.1, degrees of freedom = 10, P < 0.001. In additional analyses we compared subject scores from the 2PL model with the same subjects' scores from the 1PL model. This comparison led us to conclude that the improved fit from the 2PL model led to minimal reclassification of subjects, with respect to the subject scores estimated from the 1PL model, because overlap of 2PL scores between 1PL score categories was only observed for small numbers of outlying values.

For adult-referenced items, the difference in goodness of fit on changing from the 1PL model to the DIF model was χ^2 ^= 201.4, df = 18, P < 0.001. However, between-group differences in item calibrations, using the Afro-Caribbean group for reference, were generally very small when compared with the differences in calibration between items (Table 3). However, the item calibration for the eighth ranked item concerning 'losing weight' was significantly higher in the Afro-Caribbean group than either the Indo-Caribbean or Mixed groups. This item is close to the threshold for classification of food insecurity with severe hunger and the estimated difference might result in a downward bias in the estimated prevalence of food insecurity with severe hunger in this group. The 'balanced meal' item, ranked second in severity, showed a slightly lower calibration in Indo-Caribbean subjects when compared with Afro-Caribbean subjects.

Item calibrations for the child-referenced items from the 1PL model ranged from -3.715 (0.025) for the item concerning 'relied on a few kinds of cheaper foods' to 3.088 (0.039) for the item concerning 'children didn't eat for a whole day' (Table 2). In the 2PL model, item discrimination parameters were generally close to one. The most severe item gave the lowest discrimination parameter and this was associated with a more extreme item calibration than was obtained in the 1PL model. The improvement in goodness of fit from the 2PL model was χ^2 ^= 33.9, df 8, P < 0.001 but comparison of subject scores for the 2PL model with the 1PL model (Figure 1b) showed that there would be only minimal reclassification through application of the 2PL model. The improvement in goodness of fit from the DIF model compared with the 1PL model was χ^2 ^= 269.1, df 14, P < 0.001. When compared to the Afro-Caribbean group, Indo-Caribbean subjects showed slightly lower calibrations for the two items ranked lowest in severity with slightly higher calibrations for the fifth and sixth ranked items (Table 3). These differences were observed to a lesser degree in the group of Mixed ethnicity. These differences could lead to a slight upward bias in the estimation of food insecurity without hunger in Indo-Caribbeans with an equivalent bias in the estimation of food insecurity with hunger in the Afro-Caribbean group.

Table 4 shows the distribution of food security status of adults and children by ethnicity. In the classification for adults, food insecurity was slightly less frequent overall in subjects of Indo-Caribbean ethnicity compared to Afro-Caribbean (odds ratio 0.76, 95% confidence interval 0.57 to 1.03, P = 0.072). In the classification of children, food insecurity was as frequent in Indo-Caribbean respondents compared with Afro-Caribbean (0.86, 0.64 to 1.17, P = 0.353). Table 4 also shows the association between the food security status of children or adults and monthly household income. In both children and adults food insecurity showed a strong, graded association with lower monthly household income (P < 0.001). Food insecurity with hunger became frequent at lower levels of household income than food insecurity without hunger. After additionally adjusting for income, subjects of Indo-Caribbean ethnicity were less likely to experience food insecurity both according to the adult-referenced classification (0.61, 0.47 to 0.78, P < 0.001) and child-referenced classification (0.70, 0.54 to 0.90, P = 0.005).

## Discussion

### Comparison with other studies

The main focus of the HFSS is on the affordability of food using items grounded in qualitative research findings[17]. The strong association of food insecurity with low income, and the similarity of item calibrations between Trinidad and Tobago and the US [9,10], suggest that this approach to food security measurement is appropriate in the Caribbean, as in other settings [18,19]. The present item calibrations can be compared with those reported by Bickel et al. [9] (Table C-1, page 70) from an analysis including all 18 items. After adjusting to the same mean and standard deviation, the range of item calibrations for the adult items in US data is from -4.10 to 3.49 which is closely comparable to our findings in spite of the different estimation procedure used. Future studies, might investigate whether significant quantities of food are grown, gathered or exchanged by low-income households in different Caribbean communities but the present data generally support the HFSS model of food security for use in Trinidad and Tobago.

Some differences between our data and US data are noteworthy. The item concerning 'balanced meals' gives a lower relative severity in Trinidad and Tobago both in the present data and in previous studies [7,8] and this finding has also been confirmed in data from Brazil [19]. The 'balanced meal' item has been the subject of a number of criticisms. In qualitative data, it is apparent that there may be disagreement among different groups of respondents concerning what constitutes a 'balanced meal'[12]. In quantitative data, the 'balanced meal' item is less discriminating than the other food insecurity items [20]. This item was also less discriminating in the Trinidad and Tobago data. However, subject scores from the 2PL model gave minimal misclassification in comparison with subject scores from the 1PL model. This suggests that the improvement in goodness of fit from the 2PL model is not sufficient to justify abandoning the simpler, one-parameter model in which estimated respondent scores are a linear function of the raw score.

In previous reports, there was some evidence of differential functioning of the 'balanced meal' item according to ethnic group, with Indo-Caribbean respondents giving responses indicative of a lower relative severity for this item [7,8]. In view of this difficulty, we included an additional explanation of the 'balanced meal' concept as part of the presentation of this item in the present survey. It is of interest that the differential functioning of the 'balanced meal' item which we noted in two previous reports [7,8] was less evident in the present data. There was some evidence of differential functioning of the two least severe child-referenced items but this did not appear to be important because this difference would generally lead to a slight over-estimation of food insecurity in the Indo-Caribbean group in whom food insecurity appeared to be least frequent. However, the lower relative calibration of the fifth ranked child-referenced item might be associated with a higher estimated prevalence of food insecurity with hunger in the Afro-Caribbean group. However, at a given level of income, food insecurity appeared to be less frequent in Indo-Caribbean respondents and it is possible that this reflects underlying differences in social organisation between groups[21,22].

### Strengths and limitations

Our study was based on a large sample drawn from a representative sample of schools. The overall response rate was 74% which is comparable to similar surveys in other settings. The use of a self-administered questionnaire format led to a higher frequency of item-non-response than in interview administration of similar items, but we addressed this by using recommended methods for imputing missing values. We fitted item response theory models using marginal maximum likelihood estimation and this procedure is known to give less biased estimates, particularly for more extreme items, than joint maximum likelihood estimation. However, estimated item-fit statistics may give biased results in food security data (M Nord, personal communication). For this reason, we evaluated the fit of the one-parameter logistic model by comparing results obtained using either a two-parameter model or a differential item functioning model. While these models yielded statistical evidence of better goodness-of-fit, the consequent changes to item parameter estimates and classifications based on subject scores were modest. While item response theory models assume that items are independent, the food security module includes several dependent pairs of items. However, estimation of item calibrations after omission of dependent items showed that this had minimal influence on the magnitude of item calibrations.

## Conclusion

This study provides data for the 18-items from the HFSS module from an English-speaking middle-income country. Our results show that the items generally perform in a very similar manner to results obtained from the US Current Population Survey [10,9]. Item calibrations were mostly ranked in a similar order to the one observed in the US and departures from the assumptions of the 1PL model were generally not great enough to require revision of the classification of food security status. Based on these similar item calibrations, we can conclude that problems of food insecurity are considerably more frequent in Trinidad and Tobago than the US and this is consistent with aggregate statistics which document a lower national income [23] and greater problems of under-nutrition [2] in Trinidad when compared with the US. We have previously shown that the six-item version of the Household Food Security Scale provides satisfactory results in Trinidad and Tobago[7,8]. The present results show that when it is not essential to minimise the burden on respondents, then the 18-item scale can be used. The 18-item instrument permits a distinction to be made between moderate and severe hunger and it also allows the food security status of children and adults to be estimated separately. Future studies should therefore evaluate the usefulness of the Household Food Security Survey module for understanding the distribution and determinants of food insecurity and for developing appropriate interventions.

## Competing interests

The author(s) declare that they have no competing interests.

## Authors' contributions

The authors and Dr Deepak Mahabir were responsible for designing the study; CN and BR were responsible for implementing and supervising the fieldwork in Trinidad and Tobago; MG was responsible for data analysis and reporting; all authors approved the final paper.

## Pre-publication history

The pre-publication history for this paper can be accessed here:



## Figures and Tables

**Table 1:** Item responses and item statistics for 10 adult food security items. (1PL, one parameter logistic model; 2PL, two parameter logistic model; SE, standard error).

**Item**	**Number of item responses (3858)**	**Affirmative (row %)**	**Item-score correlation**	**1PL model Item calibration (SE)**	**2PL model Item calibration (SE)**	**2PL model Item discrimination parameter (SE)**
Worried food would run out	3744	1920 (51)	0.591	-4.082 (0.019)	-4.230 (0.020)	0.997 (0.043)
Couldn't afford balanced meals	3732	1645 (44)	0.605	-3.203 (0.017)	-3.237 (0.021)	0.603 (0.023)
Food didn't last	3756	1469 (39)	0.687	-2.622 (0.021)	-2.685 (0.019)	1.103 (0.054)
Adults skip meals	3766	755 (20)	0.782	-0.039 (0.027)	-0.148 (0.024)	1.228 (0.065)
Eat less than should	3760	794 (21)	0.781	-0.254 (0.023)	-0.255 (0.021)	1.015 (0.045)
Adults skip meals often	3741	564 (15)	0.750	0.907 (0.032)	0.911 (0.022)	1.278 (0.085)
Hungry but didn't eat	3756	458 (12)	0.705	1.547 (0.028)	1.634 (0.023)	0.970 (0.058)
Lose weight, not enough food	3680	318 (9)	0.648	2.289 (0.031)	2.504 (0.029)	0.881 (0.063)
Did not eat for whole day	3781	340 (9)	0.625	2.434 (0.034)	2.530 (0.047)	0.983 (0.081)
Adult did not eat whole day often	3777	270 (7)	0.599	3.023 (0.042)	2.975 (0.051)	1.128 (0.088)
Mean (SD)				0.00 (2.53)	0.00 (2.60)	1.02 (0.19)

**Table 2:** Item responses and item statistics for eight child food security items. (1PL, one parameter logistic model; 2PL, two parameter logistic model; SE, standard error).

**Item**	**Number of responses (3858)**	**Affirmative (row %)**	**Item-score correlation**	**1PL model Item calibration (SE)**	**2PL model Item calibration (SE)**	**2PL model Item discrimination parameter (SE)**
Few kinds cheaper foods for children	3735	1431 (38)	0.617	-3.715 (0.025)	-3.688 (0.025)	0.999 (0.049)
Couldn't feed children balanced meals	3753	1357 (36)	0.639	-3.423 (0.024)	-3.358 (0.026)	1.162 (0.079)
Our children not eating enough	3756	721 (19)	0.729	-1.275 (0.021)	-1.162 (0.023)	0.874 (0.051)
Cut size children's meals	3745	393 (10)	0.681	0.837 (0.032)	0.873 (0.032)	0.862 (0.045)
Children hungry couldn't afford food	3755	372 (10)	0.721	0.998 (0.036)	0.842 (0.029)	1.097 (0.063)
Children skip meals	3768	331 (9)	0.714	1.428 (0.042)	1.276 (0.035)	1.112 (0.079)
Children skip meals often	3751	251 (7)	0.663	2.061 (0.046)	1.874 (0.036)	1.173 (0.107)
Children didn't eat whole day	3767	165 (4)	0.514	3.088 (0.039)	3.345 (0.064)	0.798 (0.080)
Mean (SD)				0.00 (2.53)	0.00 (2.51)	1.01 (0.15)

**Table 3:** Results from Differential Item Functioning (DIF) model. Figures are difference (95% confidence interval) in food security item calibrations for Indo-Caribbean and Mixed subjects when compared to Afro-Caribbean subjects for reference.

**Adult Item**	**Difference from Afro-Caribbean item calibration (95% CI)**		**Child item**	**Difference from Afro-Caribbean item calibration (95% CI)**	
	**Indo-Caribbean**	**Mixed**		**Indo-Caribbean**	**Mixed**
Worried food would run out	0.05 (0.00 to 0.11)	0.05 (-0.01 to 0.11)	Few kinds cheaper foods	-0.19 (-0.25 to -0.13)	-0.07 (-0.13 to -0.01)
Couldn't afford balanced meals	-0.06 (-0.11 to -0.01)	0.11 (0.06 to 0.16)	Children balanced meals	-0.24 (-0.32 to -0.16)	-0.04 (-0.11 to 0.02)
Food didn't last	0.05 (-0.01 to 0.11)	0.12 (0.07 to 0.17)	Our children not eating enough	-0.04 (-0.10 to 0.01)	0.01 (-0.04 to 0.06)
Adults skip meals	0.13 (0.03 to 0.22)	0.06 (-0.02 to 0.15)	Cut size children's meal	0.05 (-0.03 to 0.15)	0.01 (-0.07 to 0.08)
Eat less than should	0.08 (0.00 to 0.16)	-0.06 (-0.12 to 0.00)	Children hungry	0.20 (0.10 to 0.30)	0.11 (0.04 to 0.19)
Adults skip meals often	0.04 (-0.05 to 0.13)	0.04 (-0.04 to 0.12)	Children skip meals	0.10 (0.01 to 0.19)	0.02 (-0.08 to 0.11)
Hungry but didn't eat	0.08 (0.01 to 0.16)	-0.02 (-0.08 to 0.05)	Children skip meals often	0.06 (-0.05 to 0.17)	-0.01 (-0.12 to 0.10)
Lose weight, not enough food	-0.43 (-0.52 to -0.34)	-0.32 (-0.40 to -0.24)	Children didn't eat whole day	0.06 (-0.04 to 0.17)	-0.02 (-0.11 to 0.07)
Not eat for whole day	0.09 (-0.02 to 0.19)	0.04 (-0.07 to 0.15)			
Adults not eat whole day often	-0.02 (-0.16 to 0.13)	-0.03 (-0.16 to 0.11)			

**Table 4:** Association of food security status with ethnicity and monthly household income. Figures are frequencies (row percent).

	**Adult-referenced classification^a^**				**Child-referenced classification^b^**		
	
	**Total**	**Food insecure without hunger**	**Food insecure with moderate hunger**	**Food insecure with severe hunger**	**Total**	**Food insecure without hunger**	**Food insecure with hunger**
**All**	3774^c^	720 (19)	374 (10)	213 (6)	3776^d^	885 (23)	351 (9)
**Ethnicity**							
Afro-Caribbean	1225	255 (21)	122 (10)	81 (7)	1222	281 (23)	137 (11)
Indo-Caribbean	1067	171 (16)	96 (9)	61 (6)	1065	253 (24)	87 (8)
Mixed	1381	270 (20)	142 (10)	66 (5)	1383	323 (23)	116 (8)
Other and not known	101	24 (24)	14 (14)	5 (5)	106	28 (26)	11 (10)
**Monthly household income (US$)**							
≤ 33	80	14 (18)	28 (35)	19 (24)	81	32 (40)	33 (41)
34–67	219	57 (26)	53 (24)	47 (21)	220	82 (37)	69 (31)
68–133	471	142 (30)	88 (18)	65 (13)	472	178 (38)	95 (20)
134–267	800	217 (27)	101 (13)	42 (5)	801	257 (32)	84 (10)
268–533	801	137 (17)	53 (7)	11 (1)	798	171 (21)	23 (3)
534–1067	561	64 (11)	12 (2)	1 (0)	558	60 (11)	6 (1)
1068–2133	279	13 (5)	3 (1)	0 (0)	280	15 (5)	2 (1)
≥ 2134	98	6 (6)	1 (1)	0 (0)	95	6 (6)	0 (0)
Not known	465	70 (15)	35 (8)	28 (6)	471	84 (17)	39 (8)

^a ^adult-referenced classification: food insecure without hunger, three to five affirmatives; food insecure with moderate hunger six to eight affirmatives; food insecure with severe hunger, nine or ten affirmatives (reference 9)

^b ^child-referenced classification: food insecure without hunger, two to four affirmatives; food insecure with hunger, 5 or more affirmatives (reference 10)

^c ^84 cases had 'not known' values for food security classification based on adult-items

^d ^82 cases had 'not known' values for food security classification based on child-items
